# Factors Affecting Utilization of Voluntary HIV Counseling and Testing Services among Teachers in Awi Zone, Northwest Ethiopia

**DOI:** 10.1155/2017/9034282

**Published:** 2017-04-23

**Authors:** Woudneh Gereme Desta, Mulusew Alemneh Sinishaw, Kelemu Dessie Bizuneh

**Affiliations:** ^1^Health Promotion and Disease Prevention Core Process, Amhara National Regional State Health Bureau, Bahir Dar, Ethiopia; ^2^Clinical Chemistry, Amhara Public Health Institute, Bahir Dar, Ethiopia; ^3^Public Health Emergency Management, Awi Zone Health Department, Injibara, Ethiopia

## Abstract

HIV/AIDS affects the basic educational sector which is the most productive segment of the population and vital to the creation of human capital. The loss of skilled and experienced teachers due to the problem is increasingly compromising the provision of quality education in most African countries. The study was proposed to determine the magnitude of VCT utilization and assess contributing factors that affect VCT service utilization among secondary school teachers in Awi Zone. A cross-sectional study design was conducted among 588 participants in 2014. Self-administered questionnaire was used to collect data. Data was analyzed using SPSS version 16, presented as frequencies and summary statistics, and tested for presence of significant association with odds ratio at 95% CI. More than half (53.6%) of study participants were tested for HIV. Those who had sexual intercourse, had good knowledge about VCT, were divorced/widowed, were in the age group of 20–29 years, and were married utilized VCT services two, three, four, three, and two times better than their counterparts, respectively. Actions targeting unmarried status, increase of educational level, and teachers with age groups above 30 years are necessary to follow their counterparts to utilize VCT service in order to save loss of teachers.

## 1. Introduction

Assumption of Human Immunodeficiency Virus/Acquired Immune Deficiency Syndrome (HIV/AIDS) as a serious health crisis in the past few decades shifted to its impact on social and economic structures especially of developing countries as it affects adult populations in the productive and reproductive age groups [1].

HIV/AIDS epidemic stage was reached globally at different times according to different risk factors that undermine general productivity and production of the household as well as community, scrotal, and national economic securities is being affected [2].

According to UNAIDS report on the global AIDS epidemic in 2010, globally in 2009 about 33.3 million people (2.5 million children) were living with HIV/AIDS; among these 2.5 million people are newly infected with the virus; in the same year 1.8 million people (260,000 children less than 15 years) died due to HIV/AIDS [3].

Sub-Saharan Africa remains hardest hit and has just over 10% of the world population but is the home of 24.7 million people (1.5 million new infections) living with HIV/AIDS. Sub-Saharan Africa constituted 71% of people living with HIV/AIDS in the world; among these women are more affected than men; at the same time 1.1 million people lost their life as a result of HIV/AIDS [3, 4].

Ethiopia is one of the highly affected countries with HIV/AIDS pandemic in the world. Since the first two reported AIDS cases in 1986, the disease has spread at alarming rate throughout the country [5].

Eastern African country Ethiopia was a home of 1,116,216 people (336,160 were eligible for antiretroviral treatment (ART)) living with HIV/AIDS out of which 72,945 (20,522 needed ART) were children less than 15 years. Due to the combined effect of poverty and HIV/AIDS, more than 5.4 million children under the age of 18 years were orphaned, out of which 855,720 (16%) lost at least one parent due to HIV/AIDS [6].

Different professionals are victims for HIV/AIDS disease in all sectors; particularly the basic education sector which is vital to the creation of human capital is also equally affected. The loss of skilled and experienced teachers due to HIV/AIDS related deaths and long term HIV/AIDS related illnesses are affecting the provision of quality education in most African countries [7].

The impact of HIV/AIDS in education sector causes multifaceted effect. Evidence obtained in 1998–2003 among teachers in Addis Ababa showed that HIV/AIDS related illnesses were the leading cause of death accounting for half of all mortalities [8].

Since the issue not only is a health concern, but also has social, economic, and political implications, the Federal Ministry of Health has undertaken various appropriate reversing measures [9]. One strategy for prevention and control of HIV/AIDS is provision of extensive voluntary HIV counseling and testing services.

Voluntary HIV counseling and testing is the process by which an individual undergoes counseling enabling him or her to make an informed choice about being tested for HIV [1]. Voluntary HIV counseling and testing allows teachers to know their HIV serostatus and, if the result is positive, prepare for treatment as well as care and support services [10].

Many people in Ethiopia living with HIV/AIDS do not know their serostatus including teachers. Various studies undertaken in Ethiopia have shown different factors that contributed to nonacceptance of voluntary counseling and testing (VCT) services; among these factors are lack of perception of being at risk, no consideration given for VCT services, fear of HIV positive results, and fear of stigma and discrimination. Another study in Bethzatha project indicated that individuals having educational status of secondary school and above, being female, and Christian religion were associated with willingness to take VCT [11–13].

The 2013 Awi Zone Health Department annual report showed that the coverage of VCT utilization was 70% [14]. However, teachers were very few in the utilization of VCT services as compared to other professionals. There was scarcity of such studies conducted in Awi Zone especially in higher professional population groups. Hence, this study was initiated to fill the knowledge gap in identifying the factors that affect the acceptability of VCT services among teachers for exerting multidirectional HIV/AIDS prevention and controlling efforts, responsible professionals for creating human capital for next generation, involving shaping of school adolescents. Furthermore, the findings of this study were intended to help policy makers and planners as an input for their future program planning by identifying factors associated with VCT utilization among secondary school teachers.

**Figure 1:**
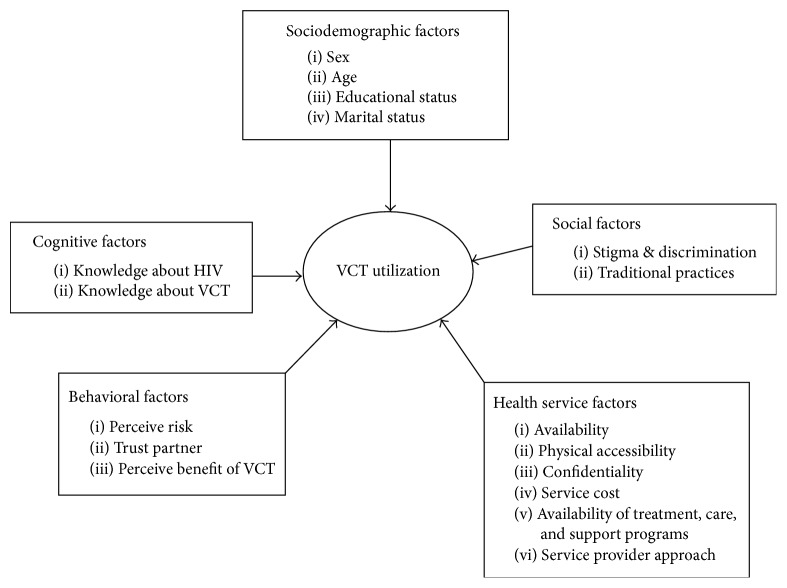
Conceptual framework for factors influencing VCT service utilization among secondary school teachers in Awi Zone, Amhara Region, 2014.

This study was intended to determine magnitude of VCT service utilization and assess the associated factors among secondary school teachers in terms of sociodemographic, cognitive, and service delivery factors (Figure 1).

*Note*. Respondents were considered to have comprehensive knowledge about HIV/AIDS if they knew about the three HIV prevention methods (abstinence, being faithful to one HIV uninfected partner, and condom use).

## 2. Methods and Materials

### 2.1. Study Period and Setting

A cross-sectional study was conducted in secondary school teachers from September to October 2014 in Awi Zone. Awi Zone, one of the eleven zones in Amhara National Regional State, located 447 km away from Addis Ababa at north west direction along Bahir Dar highway. There were 13 secondary schools (7 high schools and 6 preparatory schools) and 1004 teachers in the zone in all its 11 district administrations. The potential health service coverage by public health facilities of the zone was 98%. VCT, PMTCT, and ART services were also given in 28, 15, and 9 health centers, respectively.

### 2.2. Study Population

All secondary school teachers, who worked at the time of the study, were included in the study whereas unconsciously sick ones at the specified period were excluded.

### 2.3. Sample Size Determination and Sampling Technique

The number of study participants required for the study was determined using single population proportion formula in consideration of 95% confidence level, 5% precision, design effect of 2, 10% nonresponse rate, and 46.3% practice rate of VCT among teachers obtained from pervious study in Ethiopia [7] from a source population of 1004. The calculated final sample size was 609.

A two-stage sampling procedure was employed in order to get representative sample size from the source population. In the first stage, seven out of 13 secondary schools (6 preparatory and 7 high schools) were selected using lottery method simple random sampling technique. The determined sample size was allocated to each school proportional to the size of study population. Secondly, to pick individual participant from each selected school, lists of teachers were taken from the school Administration Office and codes were given. Then the required sample sizes were selected using systematic random sampling. To determine the *K*th value for systematic random sampling a total number of teachers were divided into each cluster sample size. The first participant was selected using lottery method simple random sampling technique.

### 2.4. Data Collection Instruments and Procedure

The data were collected using face-to-face structured questionnaire adopted from HIV/AIDS behavioral surveillance survey (BSS) [15] and demographic health survey (DHS) [16]. The questionnaire was initially prepared in English, translated to Amharic version and back to English to ensure its consistency and comparability. Pretest was conducted on 30 secondary school teachers out of the study area to prevent information contamination. After the necessary modifications were made for ambiguous questions the actual data collection had taken place among study subjects.

Two supervisors and five data collectors, who have Bachelor of Science and diploma level nurses, respectively, were recruited and trained about the process of data collection for two days and collected the data.

### 2.5. Data Management and Analysis

Precoded data were entered and cleaned using Epi-info version 3.5.1 software. The entered data were exported to SPSS version 16 software and analysis was done. The data were presented using description, tables and graphs. Presence and strength of associations between dependent and independent variables were determined using odds ratio with 95% CI. Multivariable model logistic regression was employed to control possible confounding effects among variables that had *p* value < 0.2 at bivariate regression.

### 2.6. Ethical Consideration

Ethical clearance was obtained from ethical clearance committee, Addis Continental Institute of Public Health, and University of Gondar joint MPH program. Permission letters were obtained from Amhara National Regional State Education Bureau and other substructures of education sector wrote a permission letter to study institutions accordingly. Verbal informed consent of the study participants was secured after explaining the purpose of the study. All the communications with study subjects were made with strict privacy and confidentiality. To keep confidentiality no individual identifiers were used and the collected data were put in lockable cupboard and password protected computer.

## 3. Results 

### 3.1. Sociodemographic Characteristics

A total of 588 individuals with a response rate of 96.5% participated in this study. The mean age of study subjects was 29.8 ranging from 20 to 55 years with standard deviation of 6.3. Most of the respondents were males (85.2%) and 55% were single in marital status. The majority (94%) was first degree and most (86.7%) were Christian by religion. More than half of (52.4%) respondents were Agew by ethnicity (Table 1).

### 3.2. Sexual History of Study Subjects

Four hundred eighty-nine (83.1%) reported that they ever had sex at the time of the survey. From those who had sexual experience, 248 (50.7%) reported that they had more than one sexual partner. Among them, 230 (92.7%) reported that they had used condom (Table 2).

### 3.3. Knowledge, Attitude, and Practice on VCT

**Figure 2:**
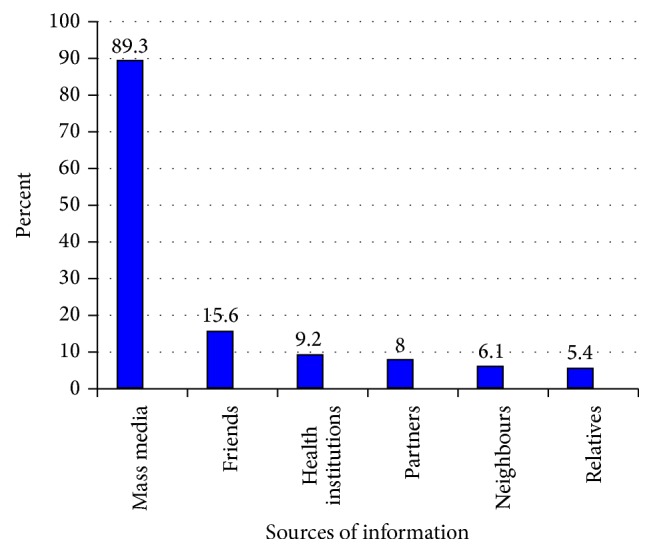
Sources of information about VCT among secondary school teachers in Awi Zone, Amhara Region, 2014.

Regarding HIV counseling and testing services all have heard about confidential VCT services. The common sources of information about HIV counseling and testing services reported by secondary school teachers were mass media 525 (89.3%) and the other 63 (10.7%) were from health institutions, friends/peers, relatives, spouse, and neighbors (Figure 2).

Of the participants who had awareness about VCT services, 575 (100%) knew that voluntary counseling and testing service is available in their localities. Of them, 425 (72%) reported that the service process could take more than 30 minutes including walking time to reach the service site while others, 163 (28%) teachers, reported that they could have got the service within less than 30 minutes (Table 3). The main sites stated by teachers to get VCT service other than their localities were the said hospital, 438 (74.5%), private clinics, 307 (52.2%), health centers, 233 (39.9%), family guidance clinics, 208 (35.4%), and mobile sites, 8 (1.4%) and those who did not know other sites to get VCT services were 40 (6.8%).

Seventy-two percent, 472 (72.4%), of the respondents had good knowledge about VCT. Almost all, 569 (96.7%), of respondents agreed that HIV counseling and testing service is important and the reasons given for the importance of services were as follows: to know one's own health status, 460 (78.2%), to protect oneself from being infected, 267 (45.5%), if positive not to transmit to others, 225 (38.3%), if positive mothers to protect the child from HIV transmission, 202 (34.4%), to choose partner, 173 (29.4%), to plan future life, 235 (40%), and if positive to get treatment, care, and support services, 7 (1.2%).

Regarding targets for HIV counseling and testing services, 147 (25%) said any sexually active person, 144 (24.5%) anyone expected to be at risk, 135 (22.9%) those who are to be married, 128 (21.8%) those people who have more than one partner, 107 (18.8%) partners of commercial sex worker, 68 (11.5%) people who are sick, and 17 (2.9%) all people.

Three hundred fifteen (53.6%; 95% CI: 49.4%, 57.7%) of study subjects ever had HIV test. From those study participants who had HIV test 83 (26.3%) underwent HIV test before three months, 60 (19.1%) before six months, 138 (43.8%) before one year, 13 (4.1%) before two years, 8 (2.5%) before three years, and 13 (4.1%) before four years. Of the HIV tested study subjects, 298 (93.9%) utilized counseling service. Among these participants 166 (56.3%), 106 (35.9%), and 29 (9.8%) had received both pre- and posttest, pretest, and posttest counseling services in that order.

About the service delivery, 281 (89.2%) study subjects who were tested stated that they were satisfied with the service; 33 (10.5%) claimed that they were not satisfied by the given service. The reasons reported for being satisfied are as follows: 180 (57.1%) quick service, 180 (34.3%) confidentiality, 111 (35%) privacy, 89 (28.3%) warm reception, 84 (26.7%) health workers competency, 118 (37.5%) brief counseling, 80 (25.4%) free service, and 31 (9.8%) referral linkage for treatment, care, and support services.

On the other hand, the reason mentioned for dissatisfaction of VCT services were, 18 (90%), no warm reception, 13 (65%), lack of confidentiality, 13 (65%), health workers competency, 7 (35%), no clear counseling service, 6 (30%), long waiting time, 5 (25%), no referral linkage for treatment and other care and support services, and, 4 (20%), lack of privacy.

### 3.4. Reasons for HIV Counseling and Testing

**Figure 3:**
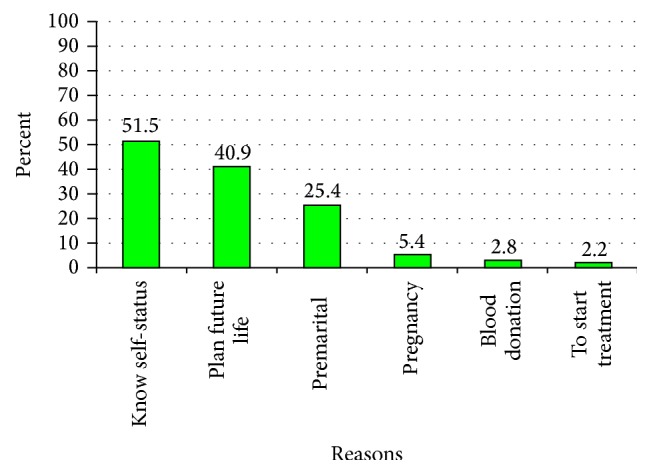
Reason mentioned to get tested for HIV among secondary school teachers in Awi Zone, Amhara Region, 2014.

**Figure 4:**
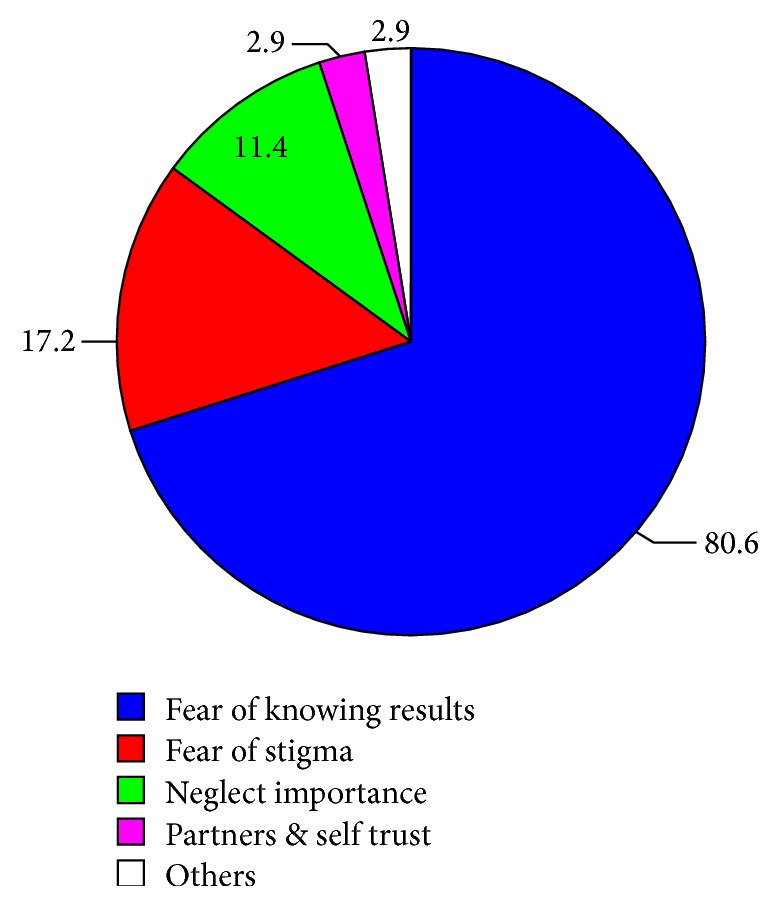
Reasons mentioned not to get tested for HIV among secondary school teachers in Awi Zone, Amhara Region, 2014.

Among respondents who utilized HIV counseling and testing services the reasons given for being tested were for knowing self-status, 161 (51.1%), for planning future life, 129 (40.9%), for premarital cases, 80 (25.4%), for blood donation, 9 (2.8%), and for starting antiretroviral treatment, 7 (2.2%) (Figure 3). On the other hand, respondents' reasons reported for not being tested were fear of result, 80.6%, fear of stigma, 17.2%, and other reasons, 17.2% (Figure 4).

### 3.5. Factors Associated with VCT Service Utilization

Respondents of age group 20–29 utilized VCT services 3 times better than 40 years and above (AOR: 3.03; 95% CI: 1.4, 6.13).

Divorced/widowed and married study participants were more than four and two times extra utilizing VCT services than unmarried counterparts (AOR: 4.38; 95% CI: 1.0, 18.32 and AOR: 2.29; 95% CI: 1.51, 3.49), respectively. In terms of education level of respondents who had first degree and above they used VCT services less as compared to the respondents of diploma and certificate holders (AOR: 0.29; 95% CI: 0.11, 0.75). However, VCT service vitalization was not significantly associated (*p* > 0.05) with sex, religion, and income of respondents (Table 4).

Respondents who had practiced sexual intercourse utilized VCT services two times greater than those who had not (AOR: 2.25; 95% CI: 1.09, 4.61). Similarly, the participants who had multiple sexual partners utilized VCT services about 2 times more than those who do not (AOR: 1.96; 95% CI: 1.37, 2.81) (Table 5). However, significant association was not observed among partners who used condom during intercourse with multiple sexual partners compared to their counterparts.

Knowledge about VCT was significantly associated with VCT service utilization. Respondents who had good knowledge utilized VCT services 3 times higher than those who had poor knowledge about VCT services (AOR: 3.05; 95% CI: 1.72, 7.21).

Respondents perceived to be discriminated by family and unwilling to disclose themselves utilized VCT services about 22% and 62% less than their counterparts (AOR: 0.22; 95% CI: 0.055, 1.01 and AOR: 0.62; 95% CI: 0.44, 0.88), respectively (Table 6).

## 4. Discussion

VCT is one of those approaches that are adopted at the national level for HIV/AIDS prevention and control strategies. This study tried to look into some of the important factors that affect the utilization of VCT among secondary school teachers in Awi Zone.

In this study we found that the proportion of VCT service utilization was 53.6%, which is still low as compared to VCT service utilized by the general community (70%) in Awi Zone [14]. This figure is considerably higher when compared to the study conducted in Harari Administrative region and the 2005 BSS-Ethiopia findings which were 46.3% and 41.3%, respectively [7, 15]. Similar study conducted among teachers in Tanzania also showed about 20% of the participants had utilized voluntary testing for HIV [17]. The increased uptake could be due to the intensive advocacy, health education, and expansion of VCT site and other related activities which have been conducted on HIV/AIDS prevention and control interventions assisted by health extension programs.

In this study, teachers aged between 20 and 29 years utilized VCT services 3 times better than 40 years and above. A study conducted among teachers in Tanzania also indicated that teachers who were aged between 21 and 30 years were significantly associated with an increased rate of having been tested for HIV [17]. Another study conducted in Kagera Region, Tanzania, also revealed an increasing tendency for young people to be tested for HIV especially before marriage [18]. Along with our findings, several population based studies conducted in Ethiopia revealed an association between testing for HIV and age; as age increases, the rate of testing for HIV decreases. The significant positive association of the younger age group may be due to the fact that they perceive themselves at risk of HIV infection.

In this study, respondents with education levels of first degree and above used VCT services less than diploma and certificate holders. This finding supported a study conducted in Gonder among different professional and community groups that showed high school teachers were found to have less acceptance of VCT compared with the other community groups [10]. This could be due to the fact that as people's knowledge increases about the serious health consequences to the extent of death and the society's reaction to the disease, people could decline to accept VCT despite good knowledge. On the other hand, the study conducted in Tanzania has showed teachers who had a college or university education have been tested for HIV greater than those who had secondary or primary education [17]. The increased uptake of VCT utilization among Tanzanian teachers might be due to the fact that in urban areas those with higher levels of education anticipated reactions of HIV/AIDS related stigma and discrimination less.

Married individuals showed utilizing VCT services 2 times more than unmarried counterparts. Moreover, those who were divorced/widowed utilized VCT services 4 times more than unmarried counterparts. Similar findings were reported from the study conducted in North Wollo Zone, Amhara Region, and in Rakai, Uganda [19, 20]. Individuals who had practiced sex ever and had multiple sexual partners were also depicted to utilize VCT services 2 times more than those who had not. Parallel finding was also reported from a study conducted among 15–49-year age group in Mersa Town of North Wollo Zone, Ambo and Bahir Dar University Students [19, 21, 22]. This might be due to the fact that those who had practiced sex with multiple sexual partners perceived that HIV is transmitted through high-risk sexual practices.

The present study indicated that individuals who had good knowledge about VCT were three times more likely to utilize VCT service than those who had not. Similar finding was reported from a study among Debre Markos University students [23]. In contrast to this, a study in Harari among teachers indicated that knowing about VCT was not significantly associated with utilization of VCT [7]. This might be because knowledgeable individuals might perceive the benefits gained from being tested for HIV at this time.

In this study individuals with discriminating attitude and fear of stigma were less likely to utilize VCT service than those who had not such perceptions. This finding is inconsistent with other study findings [19, 24, 25]. This could be due to the fact that many years of social mobilization, extensive education, and counseling bring behavioral change on stigma and discrimination attitude.

The limitation of this study was confined to one zone which may not represent other zones in the region as well as in Ethiopia.

## 5. Conclusions and Recommendation

VCT utilization rate among teachers was still low. Therefore, strengthening coordinated efforts for convincing teachers to perceive their risk behavior and to utilize VCT service is recommended with a special attention to educational level holders of first degree and above who utilized VCT less.

Focused group appropriate activities are necessary using local resources like school community conversation and HIV/AIDS clubs that will bring behavioral change regarding utilization of VCT service to sustain more utilization of VCT among age groups 20–29, those being married and windowed/divorced, those having good knowledge about VCT, and those who had ever sexual intercourse.

## Figures and Tables

**Table 1:** Sociodemographic characteristics of secondary school teachers in Awi Zone, Amhara Region, Ethiopia, 2014.

Characteristics	Category	Frequency	Percent
Sex	Male	501	85.2
	Female	87	14.8
Age group	20–29	346	58.8
	30–39	191	32.5
	40–55	51	8.7
Marital status	Single	329	55.9
	Married	249	42.4
	Widowed/divorced	10	1.7
Educational level	First degree and above	553	94.0
	Diploma and certificate	35	6.0
Religion	Orthodox Christian	510	86.7
	Muslim	58	9.9
	Others	20	3.4
Ethnicity	Agew	308	52.4
	Amhara	252	42.8
	Others	28	4.8
Monthly income	<1000 birr	12	2.0
	1000–2000 birr	352	59.9
	>2000 birr	224	38.1

**Table 2:** Sexual history of secondary school teachers in Awi Zone, Amhara Region, Ethiopia, 2014.

Characteristics	Category	Frequency	Percent
Ever had sex (*n* = 588)	Yes	490	83.3
	No	98	16.7
Ever had sex with multiple sexual partners in the last five years (*n* = 489)	Yes	248	50.7
	No	241	49.3
Ever used condom with multiple sexual partners in the last five years (*n* = 248)	Yes	230	92.7
	No	18	7.3
How often they used condom with multiple sexual partners (*n* = 230)	Always	136	58.7
	Sometimes	95	41.3

**Table 3:** Knowledge, attitude, and practice of secondary school teachers on VCT, Awi Zone, Amhara Region, Ethiopia, 2014.

Characteristics	Category	Frequency	Percent
VCT available in your locality	Yes	575	97.8
	No	13	2.2
Time to take the service	Less than 30 minutes	163	27.7
	More than 30 minutes	425	72.3
Comprehensive knowledge about VCT	Good knowledge	426	72.4
	Poor knowledge	162	27.6
VCT mentioned as important (*n* = 588)	Yes	569	96.7
	No	10	1.7
	Neutral	9	1.7
Ever had VCT test (*n* = 569)	Yes	315	53.6
	No	273	46.4
Place where HIV test was performed (*n* = 315)	Government health center	263	83.5
	Other health institutions	52	16.5

VCT: voluntary counseling and testing; HIV: Human Immunodeficiency Virus.

**Table 4:** Associations of selected sociodemographic variables with VCT service utilization among secondary school teachers in Awi Zone, Amhara Region, Ethiopia, 2014.

Characteristics	Category	Had VCT		COR (95% CI)	AOR (95% CI)
		Yes	No		
Sex	Male	266	235	0.88 (0.56, 1.39)	1.07 (0.66, 1.74)
	Female	49	38	1	1
Age group	20–29	201	145	1.69 (0.90, 3.18)	3.03 (1.40,6.13)^*∗*^
	30–39	91	100	1.11 (0.57, 2.16)	1.44 (0.76, 2.75)
	40–60	23	28	1	1
Marital status	Single	162	167	1	1
	Married	146	103	1.46 (1.03, 2.07)	2.29 (1.50,3.49)^*∗*^
	Divorced/widowed	7	3	2.41 (0.55, 11.95)	4.38 (1.05,18.32)^*∗*^
Educational level	Above first degree	286	267	0.22 (0.08,0.57)^*∗*^	0.29 (0.11,0.75)^*∗*^
	Diploma and certificate	29	6	1	1
Religion	Orthodox	278	232	1	1
	Muslim	30	28	0.89 (0.50, 1.59)	1.01 (0.58, 1.78)
	Others	7	13	0.45 (0.16, 1.23)	0.48 (0.18, 1.25)
Monthly income	<1000 birr	10	2	1	1
	1000–2000 birr	196	160	0.24 (0.04,1.21)^*∗*^	0.47 (0.077, 2.15)
	>2000 birr	109	111	0.20 (0.03,0.99)^*∗*^	0.37 (0.07, 2.05)

^*∗*^Significantly associated; VCT: voluntary counseling and testing.

**Table 5:** Association of sexual behaviors with VCT service utilization among secondary school teachers in Awi Zone, Amhara Region, Ethiopia, 2014.

Characteristics	Category	Had VCT		COR (95% CI)	AOR (95% CI)
		Yes	No		
Ever had sexual intercourse	Yes	271	219	1.52 (0.96,2.40)^*∗*^	2.25 (1.09,4.61)^*∗*^
	No	44	54	1	1
Had sex with multiple sexual partners	Yes	181	112	1.51 (1.05,2.03)^*∗*^	1.96 (1.37,2.81)^*∗*^
	No	112	136	1	1
	No response	22	25	0.54 (0.28, 1.08)	1.38 (0.53, 3.67)
Frequency of condom use during sexual intercourse with multiple partners	Yes	117	133	0.54 (0.38,0.77)^*∗*^	0.69 (0.24, 2.05)
	No	176	108	1	1
	No response	22	32	0.42 (0.22,0.79)^*∗*^	0.48 (0.27, 1.12)
Frequency of condom use with the multiple sexual partners	Always	69	66	1	1
	Sometimes	50	65	0.69 (0.41, 0.41)	0.5 (0.31, 0.8)
	No response	143	64	1.45 (0.49, 4.3)	0.5 (0.31, 0.8)

^*∗*^Significantly associated; VCT: voluntary counseling and testing.

**Table 6:** Association of knowledge, attitude, and practices towards VCT service utilization, among secondary school teachers in Awi Zone, Amhara Region, Ethiopia, 2014.

Characteristics	Category	Had VCT		Crude OR (95% CI)	Adjusted AOR (95% CI)
		Yes	No		
Educational level	Above first degree	286	267	0.22 (0.08,0.57)^*∗*^	0.16 (0.17, 2.42)
	Diploma and certificate	29	6	1	1
Knowledge of VCT	Poor knowledge	51	111	1	1
	Good knowledge	246	180	2.97 (1.99,4.45)^*∗*^	3.05 (1.72,7.21)^*∗*^
Unwillingness to disclose HIV test results for anyone	Yes	20	82	0.16 (0.09,0.27)^*∗*^	0.22 (0.055, 1.01)
	No	295	191	1	1
Attitude of discriminating by family	Yes	127	142	0.62 (0.44,0.88)^*∗*^	1.26 (0.54, 2.79)
	No	188	131	1	1

VCT: voluntary counseling and testing; HIV: Human Immunodeficiency Virus; ^*∗*^significantly associated.
